# A novel *Trypanosoma cruzi* secreted antigen as a potential biomarker of Chagas disease

**DOI:** 10.1038/s41598-020-76508-1

**Published:** 2020-11-11

**Authors:** Rana Nagarkatti, David Acosta, Nirmallya Acharyya, Fernanda Fortes de Araujo, Silvana Maria Elói-Santos, Olindo Assis Martins-Filho, Andréa Teixeira-Carvalho, Alain Debrabant

**Affiliations:** 1 Laboratory of Emerging Pathogens, Division of Emerging and Transfusion Transmitted Diseases, Center for Biologics Evaluation and Research, U.S. Food and Drug Administration, Silver Spring, MD USA; 2 Grupo Integrado de Pesquisas Em Biomarcadores, Instituto René Rachou, Fiocruz-Minas, Belo Horizonte, Brazil; 3 Departamento de Propedêutica Complementar, Faculdade de Medicina, Universidade Federal de Minas Gerais, Belo Horizonte, Brazil

**Keywords:** Infectious-disease diagnostics, Biomarkers, Biomarkers, Parasitic infection

## Abstract

Chagas drug discovery has been hampered by a lack of validated assays to establish treatment efficacy in pre-clinical animal models and in patients infected with *T. cruzi.* Reduced levels of parasite secreted antigens in the blood of infected hosts could be used to demonstrate treatment efficacy. A published proteomic study of parasite secreted antigens identified the hypothetical protein Tc_5171 as a secreted antigen. In this report, we developed Tc_5171 specific antibodies and showed that the native protein was expressed by the three life cycle stages of the parasite. Anti-peptide antibodies were able to detect the parasite antigen in blood of infected mice during the acute and the chronic phase of infection. Benznidazole treatment of infected mice significantly reduced their blood antigen levels. Of clinical significance, patients diagnosed with Chagas disease, either asymptomatic or with cardiac clinical symptoms had significantly higher Tc_5171 antigen levels compared to endemic controls. Pair-wise analysis, before and after Benznidazole treatment, of patients with asymptomatic Chagas disease showed a significant reduction in antigen levels post treatment. Taken together, our results indicate that Tc_5171 could be used as a novel biomarker of Chagas disease for diagnosis and to assess treatment efficacy.

## Introduction

*Trypanosoma cruzi (T. cruzi)*, the causative agent for Chagas disease (CD), is a blood borne parasite prevalent across various countries in Central and South America, with 13% of the Latin American population at risk of infection^1,2^. Due to the possibility of parasite transmission by blood transfusion, organ transplantation or during pregnancy, and the migration of infected individuals from endemic to non-endemic areas, CD has become a global health concern with an estimated health-care cost of 627 million USD per year^3^. *T. cruzi* infection typically presents as an acute phase, with extracellular trypomastigotes detectable in blood by microscopy and PCR, and 10–20% of the infected individuals display clinical symptoms^4^. The acute phase is followed by a lifelong indeterminate phase where no clinical symptoms may present. However, 20–30% of individuals in the indeterminate phase will develop severe symptoms associated with tissue damage in the heart and gastrointestinal tract^2,4,5^.

Only two drugs, Benznidazole (BZ) and Nifurtimox (NF) are available for the treatment of CD. Both of these drugs may induce multiple side effects in patients, such as anorexia, nausea, headaches and skin reactions, resulting in frequent early termination of the treatment^6,7^. Treatment of *T. cruzi* infected children with BZ has been shown to be effective, however, evidence for its effectiveness in treating chronic CD (CCD) is controversial^8,9^. Over decades, a decay in antibody titers to parasite antigens was observed in BZ or NF treated CCD patients, however few become seronegative^10,11^. Further, in the recently concluded BENEFIT trial, no beneficial effect of drug treatment was observed in CD patients with cardiac complications who were treated with BZ^12^. In the absence of more effective drugs, BZ is still considered the standard of care for treatment of CD. However, there is a growing consensus among researchers and clinicians that new drug development is critically needed for CD therapy.

Primary methods for the diagnosis of CD rely on the detection of anti-*T. cruzi* antibodies using serological assays^13^. Parasitological tests such as PCR and microscopy are also available, however, they are not reliable to detect low levels of parasitemia observed in the chronic phase of infection^9,14^. Seropositivity indicates exposure to the parasite but is not correlated with blood parasitemia during the chronic phase. Nevertheless, in infected individuals treated with an anti-*T. cruzi* drug, reversion from an initial seropositive to a seronegative test result could indicate cure. In contrast, a positive blood PCR test result correlates with blood parasitemia. However, due to the low sensitivity of blood PCR in the chronic phase of infection, a positive blood PCR test result obtained post drug treatment in infected individuals, indicates treatment failure but a negative result does not establish cure. Despite their limitations, these conventional assays have been used to determine the treatment efficacy of several new candidate drugs such as Posaconazole, Fexinidazole and VNI in pre-clinical animal models^15–17^.

To overcome the drawbacks of current diagnostic assays in establishing treatment efficacy, we and others have shown that it was possible to detect parasite antigens in blood of infected mice and human and discussed the use of these antigen for diagnostic applications and in pre-clinical drug development^18–21^. We reported previously the development of Enzyme Linked Aptamer (ELA) assays to detect *T. cruzi* excreted secreted antigens (TESA)^21^. The presence of these circulating parasite antigens in the blood of infected mice was indicative of ongoing *T. cruzi* infection, therefore establishing TESA as biomarkers of CD^20^. We further showed that TESA could be used to establish BZ treatment failure in mice^20^. Here we report the characterization of one of the secreted antigens and show that it is detectable in the blood of infected mice as well as in patients with CD.

## Results

### Selection and sequence analysis of the Tc_5171 *T. cruzi* excreted secreted antigen

Proteomic analysis of the secretome of infective trypomastigote life cycle stage of *T. cruzi* parasites identified antigens excreted and/or secreted by the parasite^22^. One of these antigens Tc_5171 is a hypothetical protein of unknown biological function with an estimated molecular weight of 31.61 kDa^22^. Our analysis of conserved domain using protein BLAST indicated that the C-terminal region of the protein encodes an RNA binding domain (RRM_SF) and the N-terminal region encodes a eukaryotic translation initiation factor 3 subunit G (eIF-3G) domain (see Supplementary Fig. S1A online). SignalP 4.1 analysis indicated that Tc_5171 did not encode a signal peptide for secretion. No GPI anchors were predicted in this sequence using a protozoan taxonomic set. Phylogenetic analysis showed that the Tc_5171 sequence was conserved in Trypanosomatid parasite genomes (see Supplementary Fig. S1B online). Sequence alignment indicated that percent identity ranged from 50.5% to 84.6% among the *Trypanosoma* sequences, with lower identities with *T. brucei* and other African trypanosome related species. The identity was below 32% with *Leishmania* sp. (see Supplementary Fig. S2 online). Antigenicity profile of the *T. cruzi* Tc_5171 sequence indicated that regions with higher antigenicity score generally corresponded with regions of higher homology between *T. cruzi* and *Leishmania* sequences. To limit the potential cross reactivity with *Leishmania* sp., a 11-amino acid epitope, VMRIVRALPE, with a high immunogenicity score and less than 30% identity with *Leishmania* sequences was used to generate anti-peptide antibodies in rabbits (see Supplementary Fig. S2 and Supplementary Table S1 online). Peptide VMRIVRALPE with an antigenicity score of 1.0 showed the best combination of antigenicity and sequence identity compared to Human and parasite Tc_5171 sequences (Supplementary Table S1 online).

### Expression of Tc_5171 by *T. cruzi* parasites

**Figure 1:**
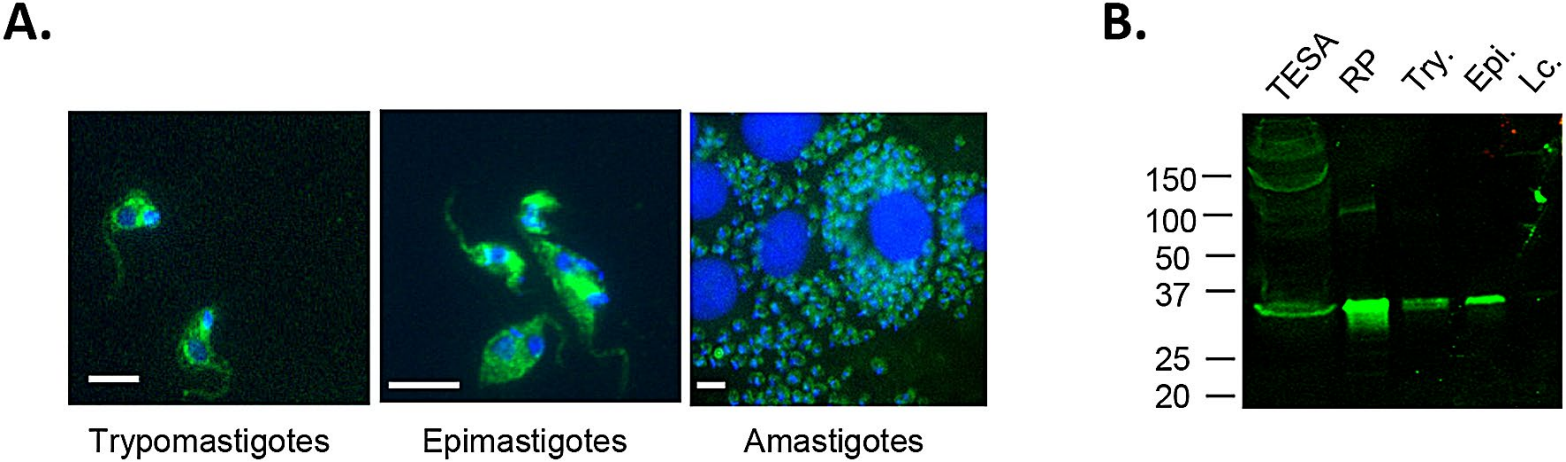
(**A**) IFA indicates that Tc_5171 is expressed by all three life cycle stages of the *T. cruzi* Tulahuen parasite, the mammalian stage trypomastigotes, the insect stage epimastigotes and intracellular amastigotes. Tc_5171 is detected using polyclonal rabbit antisera generated against the full-length protein and anti-rabbit IgG Alexa488 secondary antibody (Green). Nuclei is stained using DAPI (blue). The white bar in each panel represents 5 μm. Images were acquired using BZ-II Viewer and analyzed using BZ-II Analyzer v2.2 software (https://www.keyence.com). (**B**) Tc_5171 is detectable by western blot in TESA and migrates at a molecular weight similar to the recombinantly expressed protein (RP). The protein is detected in lysates of trypomastigotes (Try.) and epimastigotes (Epi.) but is not detectable in lysates of *L. infantum chagasi* (Lc.) parasites. Protein molecular weight ladder (in kilodalton, KDa) is represented on the left.

Codon optimized Tc_5171 sequence cloned in pET26 (b) + was expressed in *E. coli* and purified to homogeneity using the His_6_ tag (see Supplementary Fig. S3 online). A rabbit polyclonal antibody was generated against the full-length recombinant antigen. Immunofluorescence assays (IFA) with anti-Tc_5171 antibodies showed that this protein was expressed by the three life cycle stages of the parasite, trypomastigotes, epimastigotes and amastigotes (Fig. 1A). Immunohistochemistry (IHC) of heart tissue from infected C3H/HeSnJ mice showed that Tc_5171 was also expressed by in vivo *T. cruzi* amastigotes (see Supplementary Fig. S4 online). Western blots with parasite lysates confirmed that the protein was expressed by the three parasite life cycle stages and that it was secreted as a component of TESA (Fig. 1B). The Western blots also showed that the parasite protein had a molecular weight of ~ 30 kDa (Fig. 1B). No reactive band was observed in these Western blots with the *L. infantum chagasi* promastigote lysate, indicating that either the protein was not expressed by *Leishmania* or that the antibodies against Tc_5171 recombinant protein were not cross reactive. Taken together, these results demonstrated for the first time that the hypothetical protein Tc_5171, identified in the proteomic analysis of TESA, was indeed expressed by *T. cruzi* parasites.

### Characterization of the Tc_5171 anti-VMRIVRALPE peptide antibodies

**Figure 2:**
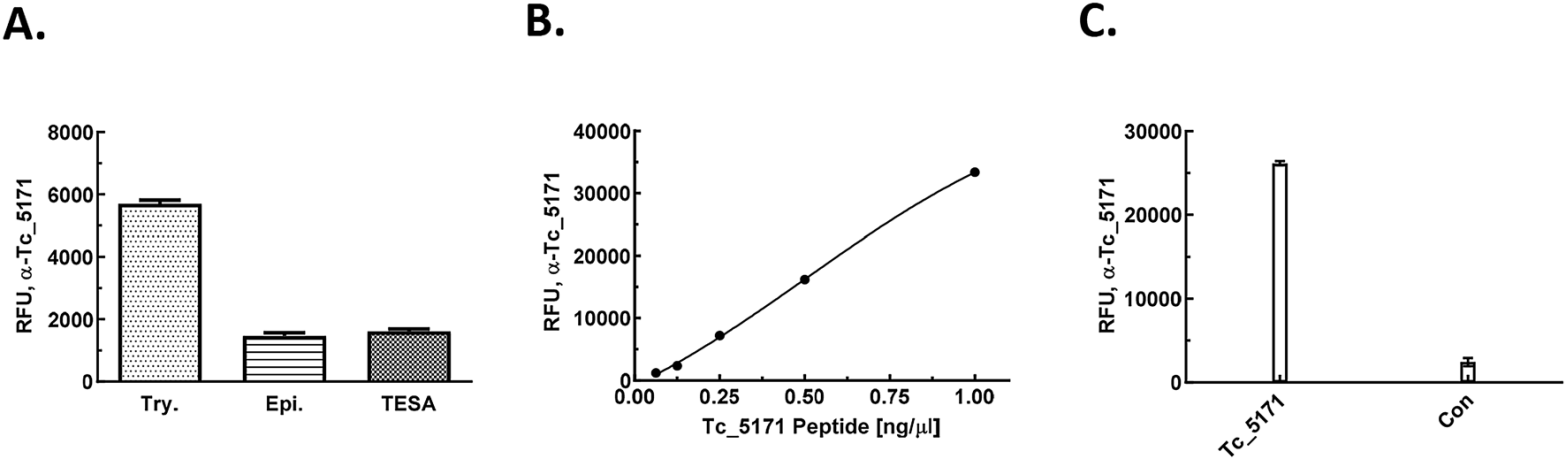
(**A**) ELISA using anti-peptide antibody detects Tc_5171 in cell extracts of trypomastigotes (Try.), epimastigotes (Epi.) and in TESA obtained from *T. cruzi* Tulahuen cultures. (**B**) ELISA using anti-peptide antibody detects up to 0.125 ng/μl of peptide spiked in diluted human plasma. (**C**) The anti-peptide antibody binds to the VMRIVRALPE peptide but not a control peptide, both peptides coated with 1 ng/µl per well diluted in 1:200 normal human plasma, by ELISA. Relative fluorescence units (RFU) obtained in the ELISA is plotted as a mean of values from duplicate wells on the Y-axis for experiments shown in panels A–C.

Antibodies from the rabbit polyclonal sera generated against the VMRIVRALPE peptide of the Tc_5171 antigen were affinity purified over a peptide conjugated sepharose column as described in the methods section. ELISA using anti-VMRIVRALPE peptide (referred as anti-peptide thereafter) antibody, performed with protein extracts from *T. cruzi* trypomastigotes, epimastigotes, and TESA preparations, showed that the anti-peptide antibody could detect the Tc_5171 antigen in the two parasite lysates and in TESA (Fig. 2A). Dose dependent binding was observed by ELISA using dilutions of the VMRIVRALPE peptide spiked in a 1:200 dilution of normal human plasma. Human plasma was used to reflect the composition of the sample matrix in the final assay configuration. The anti-peptide ELISA could detect as low as 0.125 ng/µl of the peptide spiked in plasma (Fig. 2B). Based on these results, diluted plasma spiked with the peptide at 1 and 0.1 ng/µl were used as internal positive controls to normalize the data for each ELISA plate and to allow comparison between different experiments. Further, the antipeptide antibody bound only to the VMRIVRALPE peptide coated wells and not to a control unrelated peptide, PRVRDITKRA from the *T. cruzi* flagellar calcium binding protein sequence, indicating the specificity of this interaction (Fig. 2C).

### Detection of Tc_5171 in the plasma of *T. cruzi* infected mice

**Figure 3:**
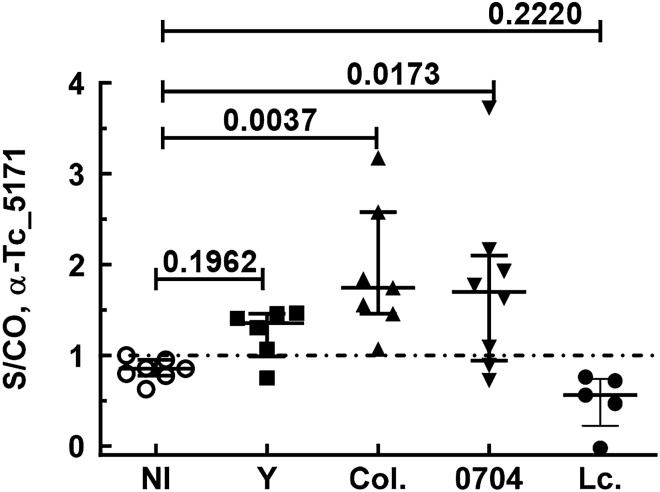
Tc_5171 antigen levels detected by ELISA in plasma obtained from mice infected with the Y strain (n = 6), the Colombiana strain (Col., n = 7) and the 0704 isolate (n = 8) of *T. cruzi* were compared with the non-infected group (NI, n = 7). Plasma from *L. infantum chagasi* infected mice (Lc., n = 5) was also tested. The S/CO for each sample is represented on the Y-axis with lines depicting the mean values ± s.d for each group. The dashed line represents the S/CO of 1 with values above 1 indicating the presence of the Tc_5171 antigen in the sample. Adjusted *p* values obtained using the nonparametric Kruskal–Wallis test are shown, with values < 0.05 indicating a significant difference between groups. Lines represent the median value with the interquartile range.

Anti-peptide antibody ELISAs were performed with plasma collected from mice infected with either, the Y, Colombiana, 0704 strain of *T. cruzi* or with *Leishmania infantum chagasi*. Results showed that mice infected with *T. cruzi* had a mean signal over cutoff (S/CO) higher than in the non-infected group indicating that the antigen was expressed and secreted in blood of mice infected by three different strains of the *T. cruzi*, although the S/CO were statistically significant (*p* < 0.05) in two of the three strains tested (Fig. 3). Homologues of the Tc_5171 sequence were found in *Leishmania* genomes; however, the anti-peptide antibody was specific to *T. cruzi* as the S/CO with *L. infantum chagasi* infected mice were below 1 like the non-infected group. Although no cross reactivity was observed with *L. infantum chagasi* proteins by western blot using antibodies generated against the recombinant full-length protein (Fig. 1B), significant cross reactivity (*p* = 0.0233) was observed with that antibody by ELISA with plasma from *L. infantum chagasi* infected mice (see Supplementary Fig. S5 online). These results indicated that using the polyclonal antibodies raised against the full-length antigen could potentially cause false positive detection of CD in *T. cruzi* and *Leishmania* co-endemic regions. Therefore, the anti-peptide ELISA was used for subsequent assays to detect the Tc_5171 antigen in mice and human blood samples.

### Tc_5171 antigen levels in plasma predict drug efficacy in acute and chronic mouse models of CD

**Figure 4:**
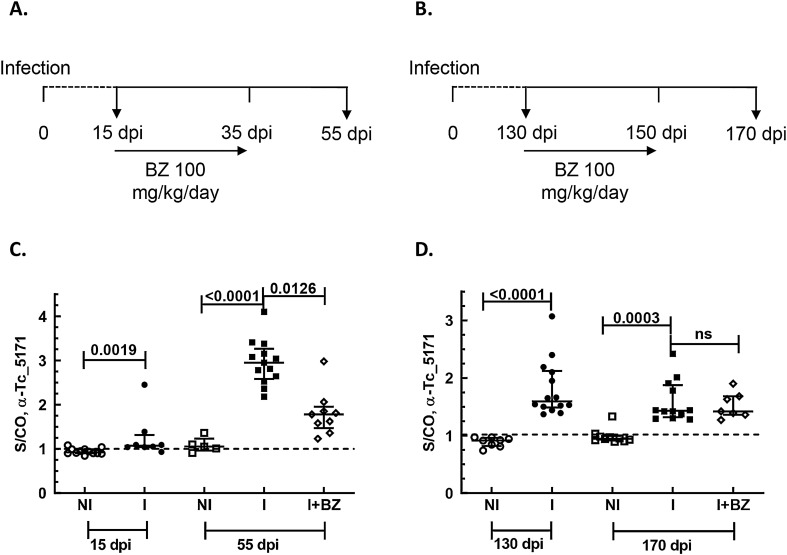
(**A**) Schematic timeline of BZ treatment schedule of Swiss mice, infected with the Colombiana strain of *T. cruzi*, during the acute phase of infection. Treatment was started at 15 dpi and continued till 35 dpi. The mice were sacrificed at 55 dpi. The arrows represent the day blood samples were collected. At 15 dpi the groups were labeled as non-infected (NI, n = 12) and infected (I, n = 8). At 55 dpi the groups are labeled as non-infected (NI, n = 5), infected (I, n = 13), and infected treated (I + BZ, n = 9). (**B**) Schematic timeline of BZ treatment schedule of C57BL/6 mice, infected with the Colombiana strain of *T. cruzi*, during the chronic phase of infection. Treatment was started at 130 days dpi and continued till 150 dpi. The mice were sacrificed at 170 dpi. The arrows represent the day blood samples were collected. At 130 dpi the groups were labeled as non-infected (NI, n = 8) and infected (I, n = 14). At 170 dpi the groups are labeled as non-infected (NI, n = 10), infected (I, n = 12), and infected treated (I + BZ, n = 7). ELISA results using the anti-peptide antibody in (**C**) and (**D**) show the S/CO, plotted on the Y-axis, with lines depicting the median values with the interquartile range for each group. The dashed line represents the S/CO of 1 with values above 1 indicating the presence of the Tc_5171 antigen. For the age matched NI and I group at 15 dpi and 130 dpi, *p* values obtained using unpaired nonparametric Mann–Whitney test are shown. For NI, I and I + BZ groups at 55 dpi and 170 dpi, adjusted *p* values obtained using the nonparametric Kruskal–Wallis test are shown. *p* values < 0.05 indicate a significant difference between groups.

To establish that Tc_5171 antigen is a suitable biomarker of CD and to validate its utility to determine treatment efficacy, it was necessary to demonstrate a correlation between the antigen level in blood and the parasite load in infected animals as determined by PCR. Anti-peptide ELISAs were performed with mice plasma samples collected at different time points after infection and BZ treatment administered either during the acute phase or during the chronic phase (Fig. 4A, B, respectively).

Swiss mice infected with the Colombiana strain of *T. cruzi* were used for the acute phase model^20^. In this model, BZ treatment was initiated at 15 dpi (Fig. 4A). ELISA performed with plasma from mice at 15 dpi yielded an average S/CO of 1.2 with 87% (7/8) of the mice in this group positive for the secreted Tc_5171 antigen compared to the age matched non-infected group (*p* = 0.0019) (Fig. 4C). As the infection progressed the levels of secreted Tc_5171 antigen increased to an average S/CO of 3.0 with 100% (13/13) mice positive by ELISA at 55 dpi compared to the age matched non-infected group (*p* < 0.0001). In the BZ treated group, the S/CO of the drug treated mice showed an average of 1.9 and was significantly lower (*p* = 0.0126) than the untreated group at the same time point (Fig. 4C). Further, the average S/CO of the drug treated group was higher than that of the age matched non-infected control group, consistent with the positive infection status of these animals, as reported previously^20^.

In the chronic phase drug treatment model, C57BL/6 mice were infected with the Colombiana strain and treated with BZ starting at 130 dpi (Fig. 4B)^20^. At 130 dpi, prior to drug treatment, the average S/CO for Tc_5171 was 1.9 with 100% (14/14) of the mice showing a S/CO above 1 compared to the age matched control group (*p* < 0.0001) (Fig. 4D). Post treatment, at 170 dpi, the average S/CO was 1.5 in the BZ treated group, which was not significantly different compared to the untreated group (Fig. 4D). However, a S/CO above 1 indicates that, except for one mouse, all other animals in the BZ treated group were still infected, also consistent with the positive infection status of these animals as reported previously^20^.

Together, the Tc_5171 anti-peptide ELISA results above show a positive correlation between the presence of Tc_5171 in blood and the infection status of the mice post BZ treatment. These results demonstrate that the secreted parasite Tc_5171 antigen is an effective biomarker of CD and show its potential use to assess drug treatment in pre-clinical mouse models.

### Tc_5171 antigen is detected in the plasma of patients with Chagas disease

**Figure 5:**
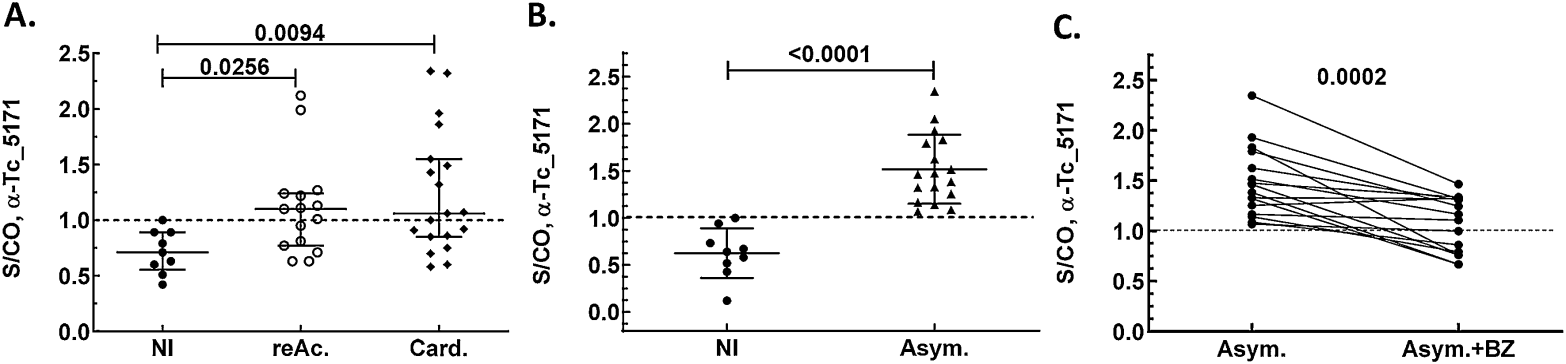
(**A**) Tc_5171 is detected at significantly higher levels by ELISA in CD patient samples from reactivated CD (reAc., n = 15) and from patients with cardiac symptoms (Card., n = 19) compared to endemic non-infected controls (NI, n = 9). (**B**) Significantly higher levels of Tc_5171 are detected in samples from patients with asymptomatic CD (Asym., n = 17) compared to the endemic non-infected controls (NI, n = 9) and upon BZ treatment the parasite antigen levels decreased significantly (Asym. + BZ, n = 16). (**C**) Pair-wise comparison asymptomatic patients before (Asym., n = 16) and after drug treatment (Asym., n = 16) shows a significant reduction in the levels of Tc_5171 antigen. ELISA results show the S/CO, plotted on the Y-axis, with lines depicting the median values with the interquartile range for each group. The dashed line represents the S/CO of 1 with values above 1 indicating the presence of the antigen. For NI, reAc. and Card. groups, adjusted *p* values obtained using the nonparametric Kruskal–Wallis test are shown. *p* values obtained using unpaired nonparametric Mann–Whitney (NI, and Asym. groups) and paired t test (Asym. and Asym. + BZ) are shown. *p* values < 0.05 indicate a significant difference between the groups.

**Figure 6:**
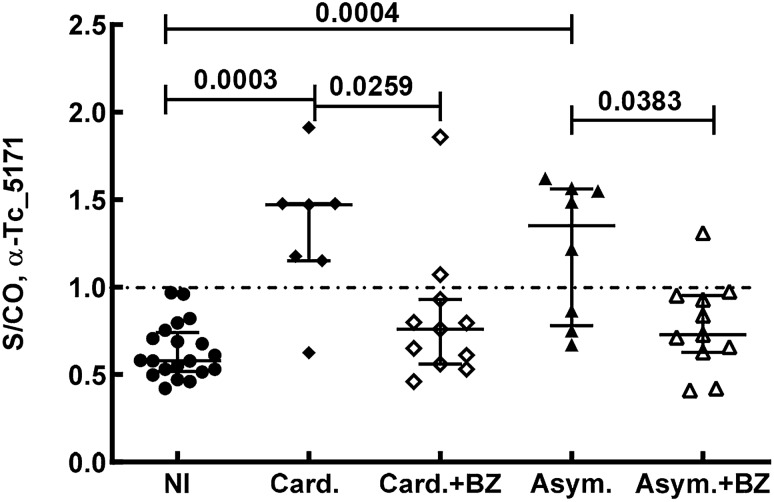
Significantly higher levels of Tc_5171 antigen is detected by ELISA in samples from patients diagnosed with cardiac symptoms due to CD (Card., n = 7) and, with asymptomatic CD (Asym., n = 8) and compared to endemic non-infected controls (NI, n = 20). Tc_5171 levels decrease significantly in both cardiac and asymptomatic patient groups after BZ treatment (Card. + BZ, n = 11; Asym. + BZ, n = 11). ELISA results show the S/CO, plotted on the Y-axis, with lines depicting the median values with the interquartile range for each group. The dashed line represents the S/CO of 1 with values above 1 indicating the presence of the antigen. Adjusted *p* values obtained using the nonparametric Kruskal–Wallis test are shown with values < 0.05 indicating a significant difference between the groups.

Blood samples from two independently collected cohorts of CD patients were obtained. Samples collected from patients with reactivated CD, with cardiac symptoms or in the asymptomatic phase (chronic asymptomatic CD) were tested with the anti-peptide ELISA to detect Tc_5171 antigen in plasma. The parasite antigen levels were significantly higher in patients with reactivated CD (9/15 with S/CO > 1, *p* = 0.0256) and in patients that exhibited cardiac symptoms (11/19 with S/CO > 1, *p* = 0.0094) compared to the non-infected endemic control group (n = 9) (Fig. 5A). All individuals in the asymptomatic group (n = 17) had S/CO > 1 and the antigen levels were significantly higher (*p* < 0.0001) than the non-infected endemic control group (n = 9) (Fig. 5B). In the asymptomatic group, individuals for whom samples were available before and after the end of BZ treatment (n = 16), a paired-wise analysis showed a significant (*p* = 0.0002) reduction in S/CO values post-treatment (Fig. 5C). Similar results were obtained in a separate independent cohort of samples collected from patients with cardiac and asymptomatic CD with a significant difference between the groups (adjusted *p* = 0.0004, Kruskal–Wallis test) (Fig. 6). In both cohorts, lower S/CO values were observed in the drug treated groups compared to non-treated individuals (Fig. 6). However, in this second cohort of CD patients, paired samples before and after drug treatment were not available from all individuals and the sample size was limited. Nevertheless, in two separate independent cohorts of patients with CD, lower Tc_5171 levels were observed in blood after BZ treatment.

Together, the anti-peptide ELISA results above show that Tc_5171 parasite antigen can be detected in blood of individuals with CD and support its utility as biomarker of human CD.

## Discussion

Biomarkers are measurable indicators of clinical disease or infection exposure used in a wide range of applications such as disease diagnosis and prognosis, evaluating drug toxicity and treatment efficacy, and in recruiting patients to clinical trials^23,24^. Limitations of the conventional *T. cruzi* diagnostic assays, such as lack of sensitivity of PCR-based assays during the chronic phase, and the lack of correlation between antibody titers and parasite load in the host, suggest that new and improved surrogate end-point assays are needed for CD drug development^8,9^. Surrogate end-points assays measuring blood biomarker levels correlating with clinical disease would provide an early indication of efficacy of treatment, compared to long term primary endpoints such as reversion to seronegativity, or improvements in cardiac functions^25^. Immunosuppression of drug treated animals has been used to allow tissue resident parasites to present as patent blood parasitemia to increase the sensitivity of PCR assays^26^. However, immunosuppression cannot be utilized to establish treatment efficacy in human clinical trials. In recent studies, serum markers of endogenous host responses such as inflammation and tissue damage induced after *T. cruzi* infection, ApoA1, NTproBNP, troponin, IFN-γ, myosin and vinculin among others, have been proposed as biomarkers of CD^27–30^. However, the lack of specificity, with respect to other health conditions where these endogenous host biomarkers may be elevated, limits their utility in clinical trials. In response to the limitations of conventional assays, a consensus Target Product Profile (TPP) for CD detection assays was developed^31–33^. To meet this TPP, assays for CD should be able to assess the efficacy of drug treatment, be easy to use in resource limited environments and be able to detect all strains of parasites.

It is well documented that the *T. cruzi* parasite antigens are released as soluble proteins, large exovesicles (100–1000 nm) and small exosomes (< 100 nm) into the extracellular milieu^22,34,35^. Levels of these parasite antigens in blood, either secreted directly by trypomastigotes, or originating from amastigotes in infected tissues (e.g., see Supplementary Fig. S4 online) and transported via the interstitial fluids to blood, would represent the overall parasite load in the infected host^19,36,37^. We have previously demonstrated that detection of TESA in blood of an infected mouse using an aptamer-based assay was as good as PCR to detect the infection and predict treatment failure^20,21^. Other reports have also indicated the utility of detecting parasite antigens in blood and urine as biomarkers for CD^38,39^.

Antigen detection assays have been well documented for parasite antigens such as HRP-II antigen of *Plasmodium falciparum* and viral antigens such as HIV p24 and the Hepatitis B surface antigen (HBsAg)^40–44^. These assays detect the residual levels of circulating antigens in blood based on antigen expression and secretion levels, and the half-life of the antigen resulting from typical biological processes such as, degradation, clearance in urine, and sequestration in immune complex or complexed with other globular proteins found in blood. Recently, ELISA using polyclonal antibodies raised against TESA and a trypomastigote lysate were used to detect antigens in plasma and serum samples obtained from patients with CD^18^. Combined with recent advances in single molecule detection using digital ELISA such as the Simoa technology, it is now possible to improve the sensitivity of antigen detection assays by 2–3 orders of magnitude over conventional ELISAs^45,46^.

However, demonstrating a correlation between parasite antigen levels in blood and the overall parasite burden in the infected host is challenging due to the existence of intracellular amastigotes in diverse tissues. Hence, we used reduction in parasite burden in response to BZ drug treatment and the expected corresponding reduction in secreted antigen levels in the blood as a method to establish and validate a new parasite secreted antigen as a biomarker of CD. In the current study, we developed an ELISA to detect the parasite secreted antigen Tc_5171 and showed a correlation between its detection in blood and the infection status of the animal as determined by blood and tissue PCR. Tc_5171 was detected is plasma of *T. cruzi* infected mice throughout the course of the infection and in the blood of mice infected with different strains of the parasite. Further, the detection of Tc_5171 could accurately predict treatment failure in acute and chronic mice models. Taken together, this Tc_5171 detection assay could be utilized for pre-clinical drug discovery with different host-parasite mice models. Of clinical relevance, this parasite antigen was also detected in plasma of CD patients and Tc_5171 levels were reduced after BZ drug treatment, further qualifying this secreted antigen as a biomarker of human CD. Although, the antigen detection assay described above fulfills some of the requirements of the CD assay TPP, additional studies using samples from well controlled clinical trials are needed to further validate Tc_5171 as a clinical biomarker of CD.

We have conclusively demonstrated, using two radically different ligands, RNA aptamers in our previous studies and anti-peptide antibodies in this report, that *T. cruzi* excreted secreted antigens such as Tc_5171, are indeed reliable biomarkers of CD. The detection of parasite biomarkers in blood represent an alternative to conventional serological and PCR-based assays for the diagnosis of CD. Parasite antigen biomarker assays also address limitations of conventional assays when used as surrogate end-point assays for drug efficacy determination and therefore represent promising new tools for preclinical and clinical applications.

## Materials and methods

### Ethics statement

Mice and human specimens were used in this study. Mice specimens were generated under an animal study protocol approved by the Center for Biologics Evaluation and Research Animal Care and Use Committee (Animal Safety Protocol #2010-03). All experiments were performed in accordance with relevant guidelines and regulations contained in the 8^th^ edition of The Guide for the Care and Use of Laboratory Animals, National Research Council, 2011 and U.S. Public Health Services Policy on animal welfare. All mice were maintained at the Center’s American Association for the Accreditation of Laboratory Animal Care accredited animal facility.

Archived human samples were originally obtained from Chagas endemic regions in Minas Gerais state in Brazil. CD was diagnosed and treated by clinical physicians at the Faculdade de Medicina, Universidade Federal de Minas Gerais, Belo Horizonte, and the Posto Avançado de Estudos Emmanuel Dias, Bambuí, Minas Gerais. All methods were carried out in accordance with relevant guidelines and regulations for human sample collection. All procedures regarding this research were approved previously by the Ethics Committee at René Rachou Institute, Fundação Oswaldo Cruz, FIOCRUZ, Minas Gerais, Brazil (Protocol #11-2004). Informed consent was obtained from all participants. Approval for the use of these archived human samples was also obtained from the Research in Human Subjects Committee at the Center for Biologics Research and Review, U.S. Food and Drug Administration (Protocol #12-102-B).

### Sequence analysis

Protein BLAST analysis, using the non-redundant protein sequence database at NCBI, was performed with the protein sequence of the putative secreted antigen Tc_5171 with a TriTryp DB i.d. TcCLB.508689.20 (https://tritrypdb.org/tritrypdb/)^47^. ClustalW sequence alignment, phylogenetic and antigenicity analysis were performed using CLC Genomics Workbench 12.0 (Qiagen)^48^. SignalP 4.1 server was used to identify potential secretory signal peptide sequence in the Tc_5171 protein sequence^49^. Amino acid motifs indicative of GPI anchors were identified using the protozoan taxonomic set (https://mendel.imp.ac.at/gpi/gpi_server.html)^50^. Immunodominant peptides from the Tc_5171 protein sequence were analyzed using the web based software SVMTriP for predicting linear B-cell epitopes in antigens (https://sysbio.unl.edu/SVMTriP/)^51^.

### Bacterial expression and purification of recombinant Tc_5171

DNA sequence encoding the putative secreted antigen Tc_5171 was codon optimized for expression in bacteria using the GenSmart codon optimization tool (GenScript, Piscataway, New Jersey, USA). The synthesized gene was cloned in pET26(b+) vector using *Nco*I and *Xho*I restrictions enzymes. The plasmid encoding the gene and the C-terminal His_6_ tag-encoding sequence was transformed into the *E. coli* BL21 (DE3) STAR expression strain. For 1 L of bacterial cell culture in minimal media, protein expression was induced at 0.8 O.D._620_ with 0.8 mM isopropyl-β-d-thiogalactopyranoside. Protein expression was performed at 37 °C with the culture flask aerated at 220 rpm on an orbital shaker for 4 h. Bacterial cells were centrifuged at 23,419×*g* (Sorvall GSA rotor) and the cell pellet subjected to sonication and lysis using 20 ml of lysis buffer (25 mM CAPS, pH 11.0, 3% Sarkosyl, 25 mM NaCl). The lysate was centrifuged at 30,597×*g* (Sorvall SS-34 rotor) for 30 min and the supernatant loaded on a HisTRAP HP NiNTA column (GE Lifesciences). The column was washed with 25 ml of wash buffer (10 mM Imidazole, 25 mM CAPS, pH 11.0, 0.3% Sarkosyl, 25 mM NaCl) and bound histidine-tagged protein eluted with a gradient of 0–350 mM Imidazole in CAPS buffer. Purified protein was dialyzed into HBS buffer (25 mM HEPES, pH 7.5 buffer with 135 mM NaCl).

### Polyclonal antibody production against the immunodominant peptide and the recombinant protein

The immunodominant peptide (VMRIVRALPE) was chemically synthesized and coupled to Keyhole limpet hemocyanin (KLH) using standard methods at the Facility for Biotechnology Resources, CBER, FDA^52^. Rabbits were immunized with 125 μg per dose of either the KLH-peptide or the purified recombinant Tc_5171 antigen, with three antigen boosts at 21-day intervals to generate polyclonal sera. Antibodies against the VMRIVRALPE peptide were affinity purified from the serum of the immunized rabbit using the SulfoLink Immobilization Kit for Peptides from Thermo Fisher. Briefly, the VMRIVRALPE peptide, synthesized with an N-terminal cysteine was covalently coupled to the iodoacetyl group on agarose beads. Peptide coupled beads were incubated with 1:2 dilution of rabbit serum in PBS. Peptide bound antibodies were eluted using 0.1 M Glycine HCl and neutralized using 1 M Tris, HCl pH 9.0. Polyclonal IgG antibodies generated against the full-length recombinant Tc_5171 antigen were purified using a HiTrap Protein G HP antibody purification column from GE Lifesciences using manufacturer recommended protocol. Purified antibodies were concentrated and dialyzed in HBS.

### Parasite cultures and antigen preparations

Epimastigotes of *T. cruzi* Tulahuen*,* Y2, Colombiana strains and 0704 isolate were cultured in LIT medium as described previously^20^. *T. cruzi* trypomastigotes were obtained from infected LLC-MK2 (ATCC CCL-7) *Macaca mulatta* monkey cell line. Culture media from 4-day infected LLC-MK2 cell monolayer were centrifuged at 2000×*g* for 10 min to pellet extracellular trypomastigotes and the supernatant concentrated using a 10 kDa Amicon concentrator to obtain the TESA fraction. *Leishmania infantum chagasi* (MHOM/BR00/MER/STRAIN2) promastigotes were grown in M199 medium containing 10% heat inactivated fetal bovine serum as described previously^53^. Parasite whole cell lysates used in ELISAs and Western blots were prepared as described before^20,21^.

### Immunofluorescence microscopy

Immunofluorescence assays were performed with trypomastigotes, epimastigotes and intracellular amastigotes of the Tulahuen strain of *T. cruzi*. Extracellular parasites and MK2 cells collected 2 days after infection were washed twice in PBS, fixed for 15 min at 4 °C with 4% para-formaldehyde, washed twice in PBS and then adhered for 10 min on a glass slide in a humid chamber. Preparations were air-dried for 5 min, permeabilized for 5 min with methanol at -20 °C, washed with PBS and blocked with 10% normal goat serum diluted in PBS for 30 min. Samples were then incubated for 30 min at room temperature with anti-Tc_5171 polyclonal antibody (85 µg/ml) diluted in PBS and containing 1% normal goat serum. Slides were washed three times with PBS and then incubated with goat anti-mouse IgG secondary antibodies coupled to Alexa Fluor 488 (Thermo Scientific; at 1:1000) in blocking buffer. Finally, samples were washed three times in PBS and mounted with VECTASHIELD (Mounting medium with DAPI; Vector Laboratories, Inc.) and examined using an inverted fluorescence phase-contrast microscope (Keyence BZ-9000, BIOREVO) with an oil immersion 100x (NA 1.4) objective lens. Images were acquired and analyzed using the BZ-II Viewer and BZ-II Analyzer v2.2 software, respectively and exported as TIFF files.

### Immunohistochemical staining of heart tissue section

C3H/HeSnJ mice (n = 4) were infected with 5000 trypomastigotes of the Colombiana strain of *T. cruzi*. After 4 weeks of infection the mice were euthanized. Heart tissue isolated from the mice were fixed in 10% buffered formalin phosphate solution and paraffin embedded according to standard procedures^54^. Slides of consecutive serial sections were prepared for immune staining. Paraffin sections were dewaxed and rehydrated prior to staining^54^. Dewaxed sections were incubated with anti-Tc_5171 polyclonal antibody (1:200 dilution) and the corresponding pre-immune rabbit serum as control. All stained sections were counterstained with Hematoxylin. All the histochemical and immunohistochemical staining was performed by Histoserv Inc, Gaithersburg, MD, USA. Slides were examined using an inverted phase-contrast microscope (Keyence BZ-9000, BIOREVO) with an oil immersion 100× (NA 1.4) objective lens. Images were acquired and analyzed using the BZ-II Viewer and BZ-II Analyzer v2.2 software, respectively and exported as TIFF files.

### Western blotting

*T. cruzi* Tulahuen trypomastigotes and epimastigotes, and *L. infantum chagasi* promastigotes obtained from cell cultures were recovered by centrifugation at 2000×*g* for 10 min. The parasite pellets were washed with PBS twice and resuspended at 2 × 10^6^ parasites in 40 µl of 1× SDS PAGE Loading buffer (Bio-Rad) with 1% β-mercapto-ethanol. The samples were boiled to lyse the parasite cells and separated on a 12% SDS-Tris/Tricine Mini-PROTEAN Precast Mini PAGE gel (Bio-Rad). The SDS-PAGE separated parasite proteins were transferred to nitrocellulose membranes using the semi-dry Transblot Turbo Transfer System (Bio-Rad). Membranes were blocked with 5% fat free milk prepared in in Tris buffer saline (TBS) for 1 h on a rocking shaker at room temperature. Rabbit polyclonal antibody raised against the full-length Tc_5171 recombinant protein was diluted 1:2500 with 0.5% fat free milk prepared in TBS containing 0.1% Tween-20 (TBS-T) and incubated with the blot for 1 h. The blot was then washed three times with TBS-T and developed with goat anti-rabbit IgG labeled with IRDye 800CW (LI-COR) as per manufacturer’s instructions. Images were captured and analyzed using the LI-COR Odyssey CLx imaging system and rendered in pseudo color (green for IRDye 800CW).

Bacterial expression and purification of the recombinant Tc_5171 protein was analyzed by SDS-PAGE stained with Coomassie blue and by western-blot using a similar method as described above except that the anti-his mouse monoclonal antibody (Invitrogen) was used to detect the recombinant protein. The blots were developed using donkey anti-mouse IgG labeled with IRDye 800CW (LI-COR) as per manufacturer’s instructions. Images for SDS-PAGE and western blots were captured and analyzed using the LI-COR Odyssey CLx imaging system and rendered in pseudo colors (red for Coomassie blue and green for IRDye 800CW).

### ELISA using antibody against the VMRIVRALPE peptide to detect Tc_5171 antigen

To detect the Tc_5171 antigen in parasite extracts and in TESA, and to establish the specificity of the antigen detection ELISA for the VMRIVRALPE peptide, 96 well Nunc Immunosorb ELISA plates were coated with 100 µL/well, in duplicate, of 200 ng/µl of either TESA or protein extracts from epimastigotes, trypomastigotes of *T. cruzi* Tulahuen strain or promastigotes of *L. infantum chagasi*. The VMRIVRALPE peptide derived from the Tc_5171 antigen sequence and an unrelated peptide (PRVRDITKRA) derived from the sequence of the *T. cruzi* flagellar calcium binding protein, used as a specificity control, were also coated at 1 ng/µl per well. To detect the Tc_5171 antigen in mice or human plasma, 100 µL/well, in duplicate of 1:200 diluted sample in PBS were coated in ELISA plates for 1 h^20,21^. The following steps were common to all anti-peptide ELISAs. Sample coated plates were blocked with 1% bovine serum albumin (BSA) in TBS for 1 h. After discarding the blocking buffer, rabbit antibody raised against the VMRIVRALPE peptide of *T. cruzi* Tc_5171 antigen was added at a concentration of 1 µg/ml in TBS-T to each well. After 1 h of incubation, the plates were washed three times with TBS-T to remove unbound and excess antibodies. A 1:5000 dilution of anti-rabbit alkaline phosphatase conjugate (Kirkegard Perry Laboratories), prepared in 0.1% BSA containing TBS-T was added to the wells and incubated for 1 h at room temperature. After washing the plate with TBS-T, bound conjugate was detected using 4-Methyllumbelliferyl Phosphate (4-MUP, Sigma). Fluorescence was measured at an excitation wavelength of 360 nm and emission wavelength of 440 nm, with a cutoff filter of 435 nm, using a Spectra Max M5 (Molecular Devices)^21^.

ELISA data was represented either as relative fluorescence units (RFU) after subtracting plate background (wells coated with blocking buffer only) for antibody characterization experiments (Fig. 3) or as a Signal to Cutoff (S/CO) ratio for experiments with infected mice and human samples (Figs. 4, 5, 6 and Supplementary Fig. S4 online). The S/CO was calculated by dividing the mean RFU value of each sample by a constant assay cutoff value. The assay cutoff value was established such that the mean S/CO of the non-infected group was < 1. The VMRIVRALPE peptide spiked at a final concentration of 1 and 0.1 ng/µl, into a 1:200 dilution in PBS of normal human plasma, was used as internal assay plate controls to normalize the data for each ELISA plate and allow comparison between different experiments. The range of S/CO for the internal positive controls was established at 6.5 ± 1.8 for 1 ng/µl plate control and at 1.2 ± 0.25 for 0.1 ng/µl plate control from 5 independent experiments. ELISA plates where S/CO of the internal positive controls were outside the specified ranges were rejected, and the ELISA repeated. Samples with S/CO values less than one were considered negative while a S/CO ≥ 1 indicated positivity for the presence of the biomarker.

For the ELISA experiments shown in Supplementary Fig. S5 online, a polyclonal serum raised in rabbit against the full-length Tc_5171 antigen was used instead of the anti-VMRIVRALPE peptide antibody. In that case, a 1:2000 dilution of the rabbit polyclonal serum prepared in TBS-T was used. The other steps for ELISA and S/CO calculations were the same as described above.

### Mice samples

Swiss mice infected with the *T. cruzi* Colombiana strain are representative of the acute phase model and C57BL/6 mice infected with the *s*ame strain are representative of the chronic phase model of CD^20,55^. Tc_5171 anti-peptide ELISA to detect secreted *T. cruzi* Tc_5171 antigen was performed using archived samples that were well characterized using microscopy, blood and tissue PCR and an Enzyme Linked Aptamer assay to detect secreted antigens^20^. Briefly, mice infected intraperitoneally (IP) with 5000 tissue culture derived *T. cruzi* Colombiana trypomastigotes were treated for 20 consecutive days with BZ (100 mg per Kg body weight per day) starting at 15 days post infection (dpi) for Swiss mice in the acute phase and at 130 dpi for C57BL/6 mice in the chronic phase of infection. Following BZ treatment, blood and tissue samples were collected from all the animals after a recovery period of 20 days^20^. These experiments were repeated three times for the two animal models. Tc_5171 anti-peptide ELISA was performed, and data presented is representative of one experiment.

Balb/C mice were infected by intra venous injection in the tail with 5000 *L. infantum chagasi* stationary phase culture promastigotes. Infection was verified in all animals by successful culture of parasites from spleens harvested at 28 dpi^56^. Plasma was isolated from blood collected by cardiac puncture of infected mice at 28 dpi. Swiss mice infected with the Y2 and Colombiana strains, and the 0704 isolate of the *T. cruzi* parasite. Plasma collected at 28 dpi was analyzed by Tc_5171 anti-peptide ELISA.

### Human samples

Plasma samples were obtained from patients diagnosed with CD by clinical physicians. The diagnosis of CD was based on seropositivity in at least two laboratorial methods (indirect IFA or ELISA test), according to the Brazilian Consensus for CD diagnosis. *T. cruzi* infection was confirmed to be positive by ELISA (CHAGATEST, Wiener Lab) and indirect IFA (IFA CHAGAS, Biomanguinhos). For chronic CD, parasitological tests were not employed due to their low diagnostic sensitivity. The etiological treatment for CD was carried out using a regimen of Benznidazole (BZ) (5 mg/kg of body weight/twice a day for 60 days, according to the Brazilian Ministry of Health regulations—Fundação Nacional de Saúde, 2005). After treatment, patients were followed by clinical, parasitological (hemoculture) and serological follow up. All patients presented negative hemoculture and positive conventional serology during treatment follow-up.

Samples from two independently collected cohorts of patients and controls were collected and analyzed using the Tc_5171 anti-peptide antigen detection ELISA. For cohort 1 (Fig. 5), CD patients were divided into three groups. Seropositive patients undergoing T-cell immunosuppressant therapy following heart transplantation with positive blood parasitemia due to immune suppression were considered as reactivated CD cases (reAc; n = 15, age ranging from 19 to 69 years). Sero-positive symptomatic patients with dilated cardiomyopathy, diagnosed by detailed clinical examination, electrocardiography, 24-h Holter examination and chest X-ray were classified as Cardiac patients (Card.; n = 19, age ranging from 33 to 56 years). Seropositive patients with no clinical symptoms were classified as asymptomatic (Asym.; n = 17, age ranging from 35 to 52 years). For the asymptomatic group, post treatment samples from the same individuals (paired samples) were collected 1 year after treatment ended (Asym. + BZ; n = 16). The control group was composed of adult seronegative blood donors from the HEMOMINAS Blood Bank Centre, Belo Horizonte, Minas Gerais, Brazil (NI; n = 9, age ranging from 18 to 47 years).

For cohort 2 (Fig. 6) individuals had similar clinical and demographic profiles as in cohort 1 and were classified into four patient groups, Card. (n = 7), Card. + BZ (n = 11), Asym. (n = 8) and Asym. + BZ (n = 11) and a non-infected control group (n = 20) from Minas Gerais state. Samples from drug treated CD patients were collected 7 years after BZ treatment. Paired samples for patients in the pre and post treatment groups were unavailable in this cohort.

### Statistical methods

Statistical analysis was performed using GraphPad PRISM 8.0.1. Data are expressed as mean ± standard deviation (SD). A *p* value of < 0.05 was considered statistically significant. Unpaired nonparametric Mann–Whitney test was performed to compare the S/CO values of two groups where indicated. For the human samples collected from asymptomatic CD patients prior to and post BZ treatment, paired t-test was performed, and two-tailed *p* values calculated. Nonparametric Kruskal–Wallis test was performed where the S/CO of the non-infected control group was compared to that of the infected and/or the infected drug treated group. Kruskal–Wallis test was performed where paired samples were not used for the ELISAs and gaussian distribution of residuals was not detected. The mean rank of each group was compared to the mean rank of the control group to estimate inter-group *p* values. Correction for multiple comparisons by controlling the False Discovery Rate was performed at Q = 0.05 using the two-stage linear step-up methods of Benjamini, Krieger and Yekutieli.

## Supplementary information

Supplementary Information.
