# Supplementary material for: A novel Trypanosoma cruzi secreted antigen as a potential biomarker of Chagas disease
Source: Sci Rep. 2020 Nov 11;10:19591. doi: 10.1038/s41598-020-76508-1 (PMC7658208; doi:10.1038/s41598-020-76508-1)
Supplement: Supplementary file 1 — Supplementary Information. [file 41598_2020_76508_MOESM1_ESM.pdf]

A Novel *Trypanosoma cruzi* Secreted Antigen as a Potential Biomarker of Chagas Disease

Rana Nagarkatti[1+*], David Acosta[1+], Nirmallya Acharyya[1], Fernanda Fortes de Araujo[2], Silvana Maria Elói-Santos[2,3], Olindo Assis Martins-Filho[2], Andréa Teixeira-Carvalho[2], and Alain Debrabant[1*]

Supplementary Figures S1 to S5 and Table S1

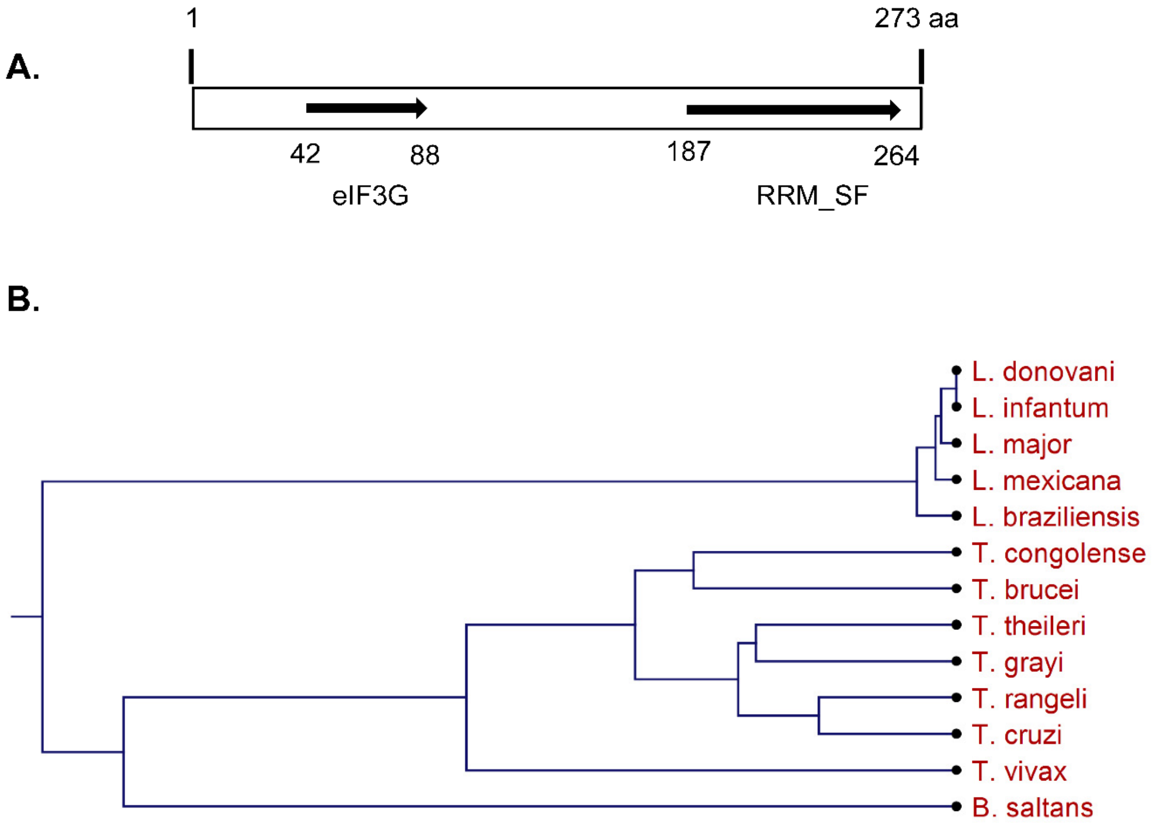

Supplementary Figure S1. (A) Schematic representation of the full-length Tc_5171 antigen (273 amino acid long). The N-terminal eukaryotic translation initiation factor 3 subunit G (eIF-3G) domain (amino acids 42 to 88) and the C- terminal RNA binding domain (RRM_SF) (amino acids 187 to 264) are indicated. **(B)** Phylogenetic analysis shows two distinct clades for sequences from Trypanosome and *Leishmania* species. *Bodo saltans* was included in the analysis as a non-parasitic predecessor to *Trypanosoma* and *Leishmania* species. Phylogenetic analysis was performed using CLC Genomics Workbench 12.0.3 (https://www.qiagenbioinformatics.com).

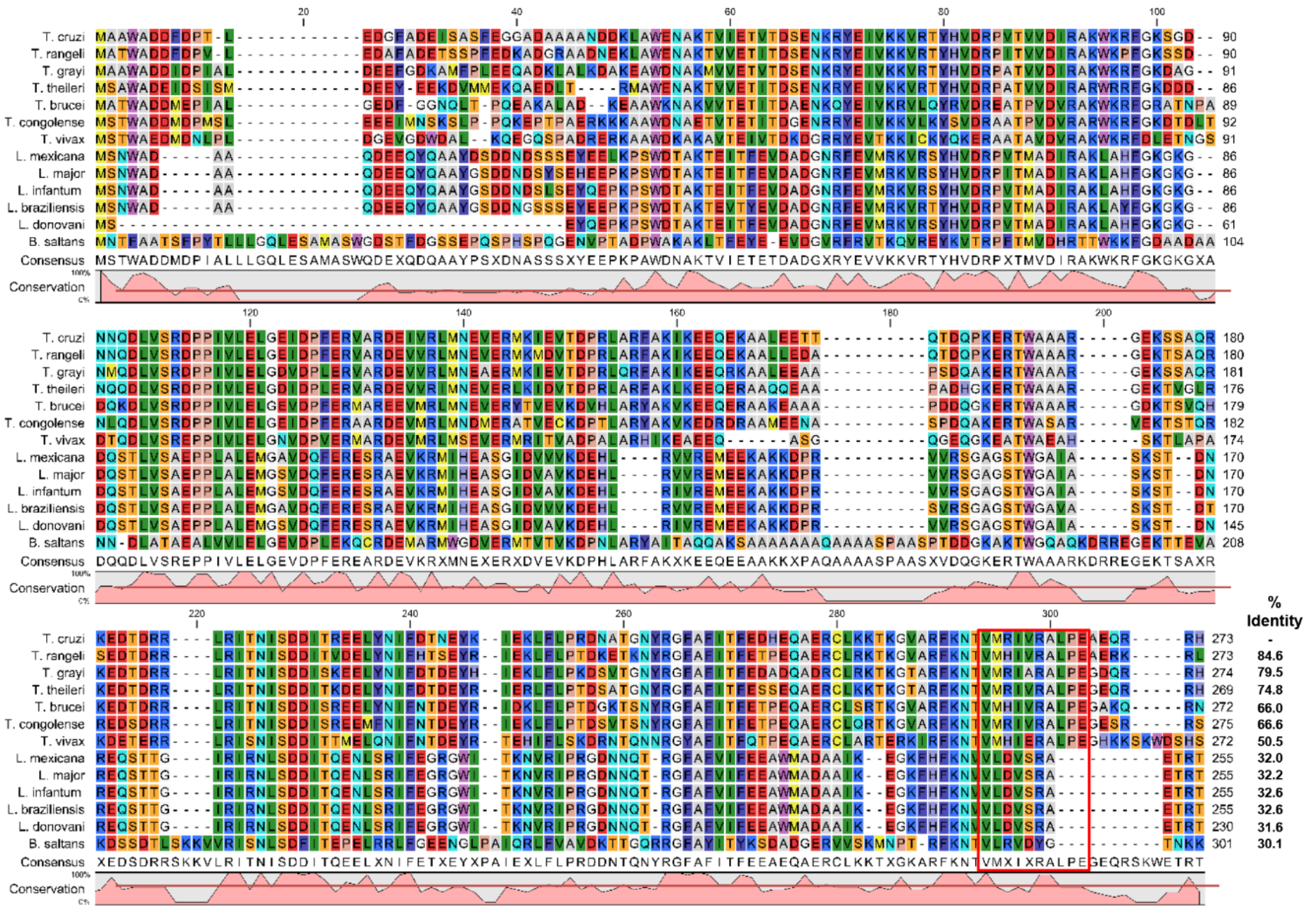

Supplementary Figure S2. CLSTALW alignment of Trypanosome and *Leishmania* sequences of Tc-5171 orthologs is shown with amino acids color based on polarity as per the RasMol scheme. Dashes indicate gaps in alignment. Percent conservation is plotted below the alignment with a line indicating 50% identity. Overall percent identity for each sequence measured against *T. cruzi* sequence is shown. The boxed region represents the peptide sequence utilized for generating anti-peptide antibodies against Tc_5171. Sequence analysis was performed using CLC Genomics Workbench 12.0.3 (https://www.qiagenbioinformatics.com).

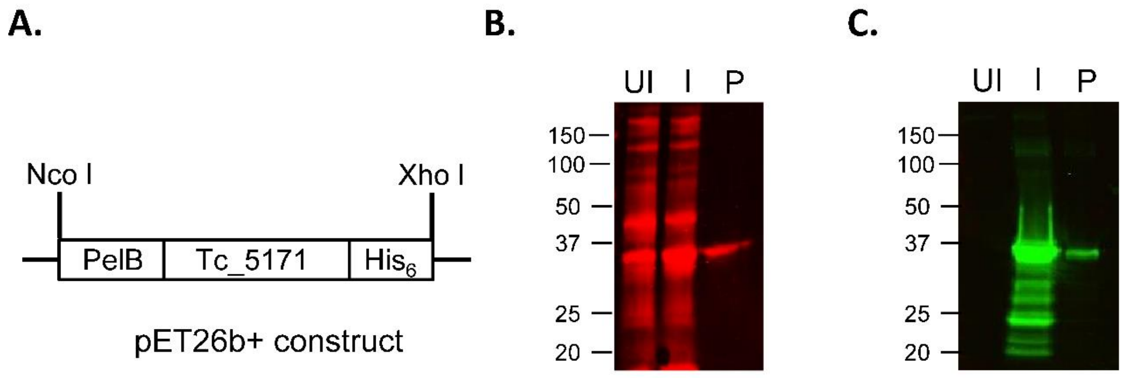

Supplementary Figure S3. (A) pET26b+ construct depicts the Tc_5171 sequence flanked by the amino terminal PelB sequence for periplasmic expression and the carboxy terminal His_6 tag for purification over a Ni-NTA column, cloned using the *Xho*I and the *Nco*I restriction enzyme sites. **(B)** *E. coli* expressed protein was purified and analyzed by SDS-PAGE stained with Coomassie blue (LI-COR pseudo color red). **(C)** Western blot of *E. coli* expressed protein using anti-his antibody (LI-COR pseudo color green). The lanes are labeled as uninduced bacterial cells (UI), IPTG induced bacterial cells (I) and purified protein (P). Protein molecular weight ladder (in kilodalton, KDa) is represented on the left.

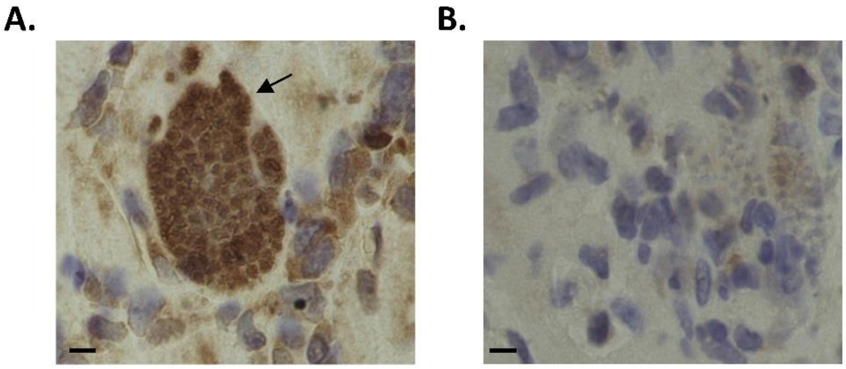

Supplementary Figure S4. (A) *T. cruzi* amastigotes (brown), stained using polyclonal antibodies raised against the recombinant Tc_5171 antigen are visible in IHC stained slides of infected heart tissue from C3H/HeSnJ mice. Tc_5171 positive amastigote nest is indicated by the black arrow (at 100x magnification). (**B)** Infected heart tissue from C3H/HeSnJ mice was stained using pre-immune sera as a control. The slides were counter stained with Hematoxylin to stain the host and parasite nucleic acid (blue). The bars represent 5 µm. Images were acquired using BZ-II Viewer and analyzed using BZ-II Analyzer v2.2 software (https://www.keyence.com). Representative image from two independent staining experiments with similar results is shown.

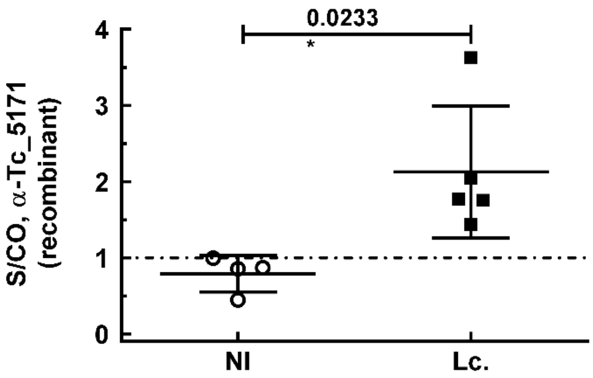

Supplementary Figure S5. Tc_5171 antigen detection ELISA, performed using polyclonal sera raised against the recombinant antigen, in plasma obtained from non-infected (NI, n= 4) and *L. infantum chagasi* infected mice (Lc., n=5). The S/CO for each sample is represented on the Y-axis with lines depicting the mean values ± s.d for each group. The dashed line represents the S/CO of 1. P values obtained using unpaired nonparametric Mann-Whitney test are shown, with values <0.05 indicating a significant difference between groups.

Rank	Location	Epitope	Score	% Identity *T. cruzi* vs Human	% Identity *T. cruzi* vs *L. donovani*
1	257-266	VMRIVRALPE	1.000	40	30
2	225-234	NYRGFAFITF	0.812	70	60
3	133-142	VTDPRLARFA	0.724	27	30
4	57-66	YEIVKKVRTY	0.668	0	50

Supplementary Table S1. Immunodominant peptides from the Tc_5171 protein sequence were identified using the web based software SVMTriP that predicts linear B-cell epitopes in antigens (http://sysbio.unl.edu/SVMTriP/). The peptides were compared to Human and *L. donovani* Tc_5171sequences to determine percent identity. Peptide VMRIVRALPE with an antigenicity score of 1 showed the best combination of antigenicity score and sequence identity with Human and parasite Tc_5171 sequences.
